# Patient participation in multidisciplinary tumor conferences: How is it implemented? What is the patients’ role? What are patients’ experiences?

**DOI:** 10.1002/cam4.4213

**Published:** 2021-08-17

**Authors:** Lena Ansmann, Christian Heuser, Annika Diekmann, Barbara Schellenberger, Claudia Biehl, Mahmoud Danaei, Christian Eichler, Dina Heinz, Andrea Hocke, Wolfram Malter, Badrig Melekian, Havva Metin, Alexander Mustea, Jenci Palatty, Uwe Peisker, Ines Petschat, Nicole Ernstmann

**Affiliations:** ^1^ Division for Organizational Health Services Research School of Medicine and Health Sciences University of Oldenburg Oldenburg Germany; ^2^ Department for Psychosomatic Medicine and Psychotherapy Center for Integrated Oncology (CIO Bonn) Center for Health Communication and Health Services Research (CHSR) University Hospital Bonn Bonn Germany; ^3^ Department of Gynecology and Obstetrics Westfälisches Brustzentrum Klinikum Dortmund gGmbH Dortmund Germany; ^4^ Breast Center Marienhospital Aachen Aachen Germany; ^5^ Breast Center Department of Obstetrics and Gynecology University of Cologne Faculty of Medicine and University Hospital Cologne Cologne Germany; ^6^ Center for Gynecological and Breast Cancer St. Mary Hospital Siegen Siegen Germany; ^7^ Department of Gynecology and Gynecological Oncology University Hospital Bonn Bonn Germany; ^8^ Clinic of Gynecology, Obstetrics and Senology Breast Cancer Center Aachen – District of Heinsberg Hermann‐Josef‐Hospital Erkelenz Germany

**Keywords:** breast cancer, decision‐making, multidisciplinary team meeting, patient participation, Tumor board, tumor conference

## Abstract

**Background:**

Prior research has shown that around 5%–7% of patients in breast cancer centers in Germany participate in the discussion of their own case within a multidisciplinary tumor conference (MTC). The PINTU study is one of the first to research this practice. The objective is to describe (a) how patient participation in MTCs is implemented, (b) what is the role of patients, and (c) how patients experience MTCs.

**Methods:**

MTCs in six breast and gynecological cancer centers in North Rhine‐Westphalia, Germany, with and without patient participation, are studied prospectively by (non)participatory, structured observation. Breast and gynecological cancer patients completed surveys before, directly after, and 4 weeks after MTC participation. Data are analyzed descriptively.

**Results:**

Case discussions of a sample of n = 317 patients (n = 95 with MTC participation and n = 222 without) were observed. Survey data were obtained from n = 242 patients (n = 87 and n = 155). Observational data showed heterogeneity in the ways MTC participation was practiced. Among participating patients, 89% had the opportunity to express their opinion and 61% were involved in decision‐making. Whereas most patients reported positive experiences and would recommend participation, some had negative experiences and regretted participating.

**Conclusions:**

Due to a lack of recommendations, hospitals implement patient participation in MTCs in many different ways. So far, it is unknown which setting and procedures of MTC participation are beneficial for patients. However, existing evidence on communication in cancer care together with this exploratory study's findings can build the basis for developing recommendations for hospitals that invite their patients to MTCs.

**Clinical trial registration number:**

German Clinical Trials Register Nr. DRKS00012552.

## Graphical abstract

Patient participation in multidisciplinary tumor conferences is a seldom, but constant reality in breast cancer care and the way it is currently practiced shows large variation. Whereas most patients reported positive experiences and would recommend participation, some had negative experiences and regretted participating. Practice recommendations for hospitals that invite their patients to tumor conferences need to be developed.

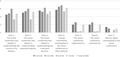

## 1 INTRODUCTION

Multidisciplinary tumor conferences (MTCs) have been widely established as a multidisciplinary teamwork tool for dealing with the complexities of cancer care. MTCs are regularly scheduled meetings of a multidisciplinary treatment team in which the diagnosis and treatment of individual cancer patients are discussed and a treatment recommendation is given.^1^ The benefits of MTCs regarding treatment decision‐making and patient outcomes have been demonstrated.^2^, ^3^, ^4^, ^5^ However, the ways in which MTCs are organized vary widely across and within countries.^4^, ^6^

In Germany, about 80% of all incident breast cancer cases in 2019 were treated at a certified breast cancer center.^7^ MTCs are a mandatory requirement for nationwide certification by the German Cancer Society, but the requirements do not address patient participation. However, the criteria of the Medical Council of the federal state of North Rhine‐Westphalia––the certifying body for that state––state that patients should be allowed to participate in the MTC if they wish to. Previous research in certified breast cancer centers throughout Germany revealed that 5%–7% of patients have participated in the MTC during their own case discussion.^1^, ^8^, ^9^ Although patient participation in MTCs is not a standard procedure, it seems to be a reality in breast cancer care in Germany. Moreover, calls demanding more patient‐centered decision‐making in MTC increase internationally.^10^

Internationally, research on benefits and risks of patient participation in MTCs is sparse, and it is unknown how patient participation is implemented. Research from Australia reveals that around 5% of all MTCs surveyed reported to always involve patients.^11^ When confronted with the idea of patient participation in MTCs, most physicians did not support it, whereas patient advocates and breast care nurses were predominantly supportive.^12^, ^13^ Another Australian study piloted patient participation in MTCs with n = 30 patients and found that most patients found it helpful and would recommend it to other patients. However, only half of the healthcare providers interviewed supported patient participation,^14^ which reflects the difficult weighing of advantages and disadvantages on this topic.

Previous research on MTC participation in breast cancer care in Germany provides further insights into patient participation. Patient surveys revealed that the participation in MTCs is dependent on patient characteristics such as health literacy and treatment regimen.^1^, ^8^ We also found that whether patients are allowed to participate or are even actively invited to do so^1^, ^8^ is highly dependent on the individual breast cancer center. An analysis of open‐ended survey questions indicated that the majority of patients experienced participation as informative and supportive, but some patients described difficult experiences and negative emotional reactions.^9^ Qualitative interviews on the perspective of healthcare providers also revealed a mixed picture of met, unmet, and disappointed needs of participating patients and their emotional reactions.^15^ This indicates that patient participation in MTCs may not be exclusively of benefit for patients. Qualitative provider interviews also gave insights into the opportunities and limits of patient involvement in MTCs^16^ and showed that only some of the central steps of shared decision‐making (SDM) can be partly implemented in the MTC under certain conditions. The provider interviews also revealed that patient participation in MTCs is predominantly regarded as being feasible for selected patients, but not in routine cancer care.^17^ Existing research still lacks insights into how patient participation in MTCs is organized and implemented in breast cancer centers in Germany. Our previous research indicated that centers differ widely in their implementation of patient participation, which may result in patients having different roles in the MTC and reporting different experiences after the MTC. Thus, a deeper insight into the practice of patient participation in MTCs, the varying ways of its implementation, and the patients’ experiences are needed to eventually enable the development of recommendations on how patient participation can be practiced to make MTCs more patient‐centered. This paper aims to answer the following three research questions: (a) how is patient participation in MTCs implemented, (b) what is the role of patients in the MTC, and (c) how do patients experience the MTC?

## 2 MATERIALS AND METHODS

### 2.1 Study design and sample

This analysis is part of a multicenter observational mixed methods study. The Patient involvement in multidisciplinary tumor conferences in breast cancer care – an exploratory study (PINTU) study has been conducted in n = 6 breast and gynecological cancer centers in North Rhine‐Westphalia, Germany.^18^ One of the cancer centers was a cooperation of two hospitals with two separate MTC meetings, resulting in n = 7 hospitals studied. The project was funded by the German Cancer Aid, received approval from the ethics committee of the Faculty of Medicine of the University of Cologne, Germany, and conforms with the Declaration of Helsinki. Data were collected between November 2018 and February 2020. Patients who were diagnosed with breast cancer or gynecological cancer (ICD codes C50.xx ‐ C58.xx, D05.xx ‐ D07.xx) and either did or did not participate in the MTC were recruited by healthcare providers in the participating centers. Before being discharged, eligible patients provided written consent. The first survey (T0) was handed out during the hospital stay and prior to the MTC, the second survey (T1) was given only to participating patients directly after the MTC, and the last survey (T2) was sent via post to all patients 4 weeks after the MTC, with two reminders. Furthermore, observations of all MTC meetings with and without patient participation were conducted by the research team. Passive participatory observations were conducted by two researchers present in the MTC, and non‐participatory observation was undertaken by three researchers using video data from the MTCs. MTCs were documented by either audio or video recordings, and each observer additionally used a structured observation protocol. Data from the observations and survey data form the basis for this analysis as one part of the larger PINTU study.

### 2.2 Instruments

In the following, the instruments used to assess the variables of interest for this analysis are described. The structured observation of case discussions in MTCs with and without patient participation included documentation of the duration of each case discussion, the number of persons present, and the seating arrangement (theater, U‐shape, or round table).

The patient surveys included validated instruments and self‐developed items (see Table 1 for the items analyzed). Survey development was informed by literature, previous interviews with healthcare providers, standards of survey development, and cognitive pretesting interviews.^18^ The T1 survey was completed only by the participating patients directly after the MTC (see Results section for detailed information). Patients’ reports on the implementation of MTC participation included information on accompanying persons, the patient's opportunity to express opinions, and patient involvement in the treatment decision. Seven self‐developed single items assessed patient experiences with participation on a 5‐point Likert scale: helpfulness in understanding the course of the disease, treatment options and treatment decision, recommendation to other patients, fear, confusion, and regret. The extent to which SDM was practiced in the MTC from the patient's perspective was assessed with the German version of the 9‐item Shared Decision‐Making Questionnaire (SDM‐Q‐9), a validated and widely established instrument.^19^ Patients were asked to relate those items to their case discussion in the MTC (see Table 1). The nine items were to be answered on a 6‐point Likert scale from 1 “completely disagree” to 6 “completely agree.” The items were summed up and divided by the number of items (Cronbach's alpha 0.891). The raw score of the instrument was converted to a score between 0 and 100.^19^

**TABLE 1:** Survey instruments used in patients with MTC participation at T1

Instrument/Topic	Items	Responses	Origin
Implementation of patient participation in MTC	Were you accompanied by someone (e.g., spouse, relative)?	Yes/no	Self‐developed, applied in previous survey of breast cancer patients in MTC in Germany^9^
	In the tumor conference, did you have the opportunity to express your opinion on further treatment?	Yes/no	
	In the tumor conference, were you involved in the decision on your further treatment?	Yes/no	
Shared decision‐making, SDM‐Q−9	In the multidisciplinary tumor conference, the treatment team made clear that a decision needs to be made.	6‐point Likert scale from 1 “completely disagree” to 6 “completely agree”	SDM‐Q−9 instrument validated for the doctor–patient consultation in diverse patient groups including breast cancer patients, languages, and countries^19^ (see www.sdmq9.com); adapted to MTC situation by replacing “my doctor…” with “In the multidisciplinary tumor conference, the treatment team…”; not applied in MTC context before
	In the multidisciplinary tumor conference, the treatment team wanted to know exactly how I want to be involved in making the decision.		
	In the multidisciplinary tumor conference, the treatment team told me that there are different options for treating my medical condition.		
	In the multidisciplinary tumor conference, the treatment team precisely explained the advantages and disadvantages of the treatment options.		
	In the multidisciplinary tumor conference, the treatment team helped me understand all the information.		
	In the multidisciplinary tumor conference, the treatment team asked me which treatment option I prefer.		
	In the multidisciplinary tumor conference, the treatment team and I thoroughly weighed the different treatment options.		
	In the multidisciplinary tumor conference, the treatment team and I selected a treatment option together.		
	In the multidisciplinary tumor conference, the treatment team and I reached an agreement on how to proceed.		
Patient experiences with MTC participation	The tumor conference was helpful in understanding the course of my disease.	5‐point Likert scale from 1 “completely disagree” to 5 “completely agree”	Newly developed
	The tumor conference was helpful in understanding treatment options.		
	The tumor conference was helpful in understanding the treatment decision.		
	I would recommend participation in the tumor conference to other patients.		
	The tumor conference frightened me.		
	The tumor conference confused me.		
	Did you regret participating in the tumor conference?		

Socio‐demographic data, such as age, living with a partner, level of education, employment status, and health insurance status were assessed with standardized factual questions at T0. Clinical data on cancer entity and UICC or FIGO staging were derived from the MTC and/or its documentation. Cancer treatment received after the MTC was assessed by self‐report at T2. Health literacy was assessed at T0 using the German version of the HLS‐EU 16^20^, ^21^ and is used in this analysis to describe the sample. The instrument assesses the four dimensions of general health literacy (accessing, understanding, appraising, and applying health‐related information). The 16 items were to be answered on a 4‐point scale from 1 “very difficult” to 4 “very easy” (Cronbach's alpha .845). All 16 items were dichotomized and summed up.^22^ Cut‐off scores were applied as recommended: 13–16 “sufficient,” 9–12 “problematic,” and 1–8 “inadequate.”

### 2.3 Analysis

Observational and survey data are analyzed descriptively and stratified by hospital, where applicable, to reflect the heterogeneity in the implementation of patient participation in MTCs. No statistical testing was applied due to the low case numbers of patients with MTC participation when stratified by hospital and due to the explorative nature of this study. Missing data were listwise deleted.

## 3 RESULTS

### 3.1 Sample description

**FIGURE 1:**
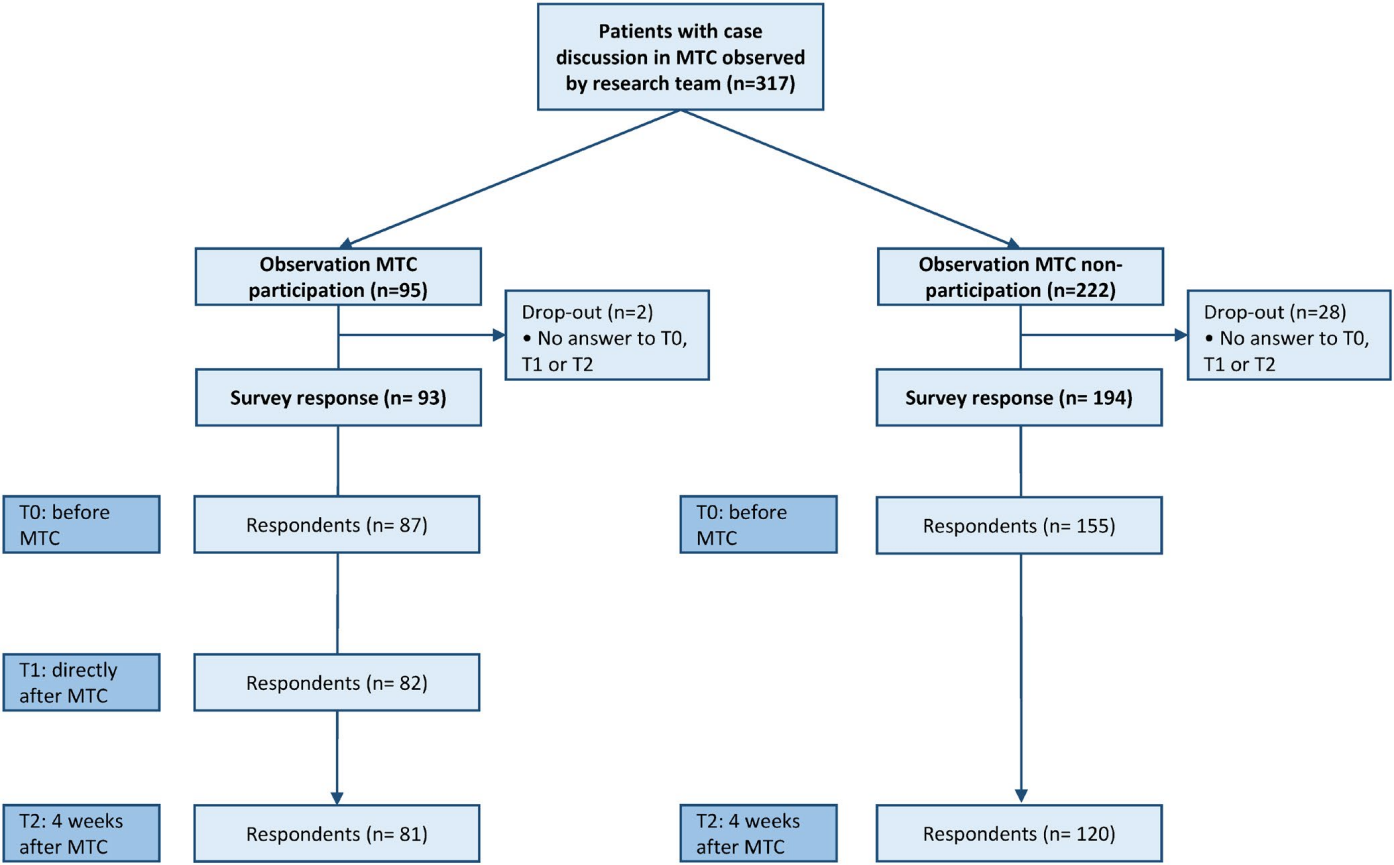
Flow of participants, divided into MTC participation and non‐participation

The study included n = 317 patients from 7 hospitals; n = 95 with patient participation and n = 222 without. Observational data from MTCs were available for all study participants. A total of n = 242 patients participated in the survey at T0; n = 87 with patient participation and n = 155 without (see Figure 1 for dropouts at T1 and T2).

Surveyed patients have a mean age of 59.9 years (see Table 2) and are predominantly patients with breast cancer (59.9%), but also patients with gynecological cancer (14.0%) or a combination (26.0%). The sample included patients in seven hospitals, of which three hospitals did not implement patient participation in MTCs. Sample characteristics are available only for the sample of n = 242 surveyed patients. However, we were able to retrieve UICC/FIGO staging and cancer entity data for the complete sample of n = 317 patients. No substantial differences between both samples were found.

**TABLE 2:** Characteristics of survey samples at T0 (n = 242), n = 87 with participation, n = 155 without participation

		MTC participation n (%)	MTC non‐participation n (%)	Total n (%)
Age (years), n = 239	*Mean (SD)*	59.1 (10.9)	60.3 (11.7)	59.9 (11.4)
Highest level of school education, n = 233	*No lower secondary school education*	1 (1.2)	2 (1.4)	3 (1.3)
	*Lower secondary school education*	21 (24.7)	45 (30.4)	64 (28.3)
	*Intermediate secondary school education*	24 (28.2)	45 (30.4)	69 (29.6)
	*University entrance certificate*	39 (45.9)	56 (37.8)	95 (40.8)
Living with partner, n = 237	*Yes*	58 (67.4)	106 (70.2)	164 (69.2)
	*No*	28 (32.6)	45 (29.8)	73 (30.8)
Currently employed, n = 196	*Yes*	25 (35.2)	43 (34.4)	68 (34.7)
	*No*	46 (64.8)	82 (65.6)	128 (65.3)
Health insurance status, n = 238	*Statutory*	67 (77.9)	117 (77.0)	184 (77.3)
	*Statutory with additional private insurance*	12 (14.0)	19 (12.5)	31 (13.0)
	*Private*	7 (8.1)	16 (10.5)	23 (9.7)
Cancer entity, n = 242	*Breast cancer*	81 (93.1)	64 (41.3)	145 (59.9)
	*Gynecological cancer*	0 (0)	34 (21.9)	34 (14.0)
	*Breast and gynecological cancer*	6 (6.9)	35 (22.6)	41 (16.9)
	*Gynecological and gastrointestinal cancer*	0 (0)	22 (14.2)	22 (9.1)
UICC staging, n = 201	*Stage 0*	10 (12.3)	8 (6.7)	18 (9.0)
	*Stage 1*	39 (48.1)	62 (51.7)	101 (47.6)
	*Stage 2*	19 (23.5)	29 (24.2)	48 (23.9)
	*Stage 3*	2 (2.5)	10 (8.3)	12 (6.0)
	*Stage 4*	11 (13.6)	11 (9.2)	22 (10.9)
Treatment received (surveyed at T2) n = 201, multiple answers possible	*Surgery*	77 (95.1)	104 (86.7)	221 (90.0)
	*Chemotherapy*	25 (20.8)	44 (36.7)	85 (34.3)
	*Radiation*	55 (67.9)	54 (45.0)	136 (54.2)
	*Endocrine therapy*	41 (50.6)	51 (42.5)	117 (45.8)
	*Do not know*	0 (0)	4 (3.3)	5 (2.0)
Health literacy, n = 231	*Inadequate*	19 (21.8)	33 (22.9)	52 (22.5)
	*Problematic*	37 (42.5)	67 (46.5)	104 (45.0)
	*Sufficient*	31 (35.6)	44 (30.6)	75 (32.5)
Hospital, n = 242	*1 (no MTC participation)*	0 (0)	36 (23.2)	36 (14.9)
	*2 (no MTC participation)*	0 (0)	35 (22.6)	35 (14.5)
	*3 (no MTC participation)*	0 (0)	22 (14.2)	22 (9.1)
	*4*	24 (27.6)	3 (1.9)	27 (11.2)
	*5*	47 (54.0)	1 (0.6)	48 (19.8)
	*6*	10 (11.5)	23 (14.8)	33 (13.6)
	*7*	6 (6.9)	35 (22.6)	41 (16.9)

### 3.2 How is patient participation in MTCs implemented?

Observations of n = 317 MTC case discussions revealed a heterogeneous picture of the practical implementation of patient participation in the hospitals examined (see Table 3). Four types were identified: (a) Non‐participation: the patient's case is discussed by healthcare providers in the MTC without the patient being present, and a treatment recommendation is developed (three hospitals). (b) The patient's case is discussed by healthcare providers in the MTC, and a treatment recommendation is developed. The patient is present throughout the case discussion (one hospital). (c) The patient's case is first discussed by healthcare providers in the MTC without the patient being present. After the treatment recommendation is developed, the patient attends the MTC, with the complete MTC team being present (two hospitals). (d) The patient's case is first discussed by healthcare providers in the MTC without the patient being present. After the treatment recommendation is developed, a smaller group of MTC members meets with the patient in a separate room to inform her about the recommendation (one hospital). We defined the first type as non‐participation and the latter three as patient participation. Due to substantial between‐hospital differences in the types of patient participation, results will be stratified by hospital where possible.

**TABLE 3:** Descriptive results from structured observations of MTC meetings, P = with patient participation, NP = without patient participation, % for seating arrangement adds up to more than 100% due to many meetings changing arrangements within the MTC, n.n. = no valid answers due to the low case numbers in NP sample in hospitals 4 and 5

Hospital	Way in which patient participation in MTC is practiced, if at all	Mean duration of case discussion in minutes, mean (SD), n = 256		Mean number of persons participating in MTC, mean (SD), n = 259		Seating arrangement, n (%)					
						Theater		U‐shape		Round table	
		P	NP	P	NP	P	NP	P	NP	P	NP
1	No participation	—	4.0 (1.1)	—	14 (2)	—	48 (100)	—	0 (0)	—	0 (0)
2	No participation	—	1.5 (0.6)	—	14 (3)	—	60 (100)	—	0 (0)	—	0 (0)
3	No participation	—	2.7 (1.0)	—	19 (5)	—	21 (95)	—	1 (5)	—	0 (0)
4	Patient invited to MTC round after case discussion to explain recommendation	9.0 (5.6)	n.n.	14 (3)	18 (0)	0 (0)	0 (0)	25 (45)	30 (55)	0 (0)	0 (0)
5	Patient invited after case discussion to another room with smaller group of MTC participants to explain recommendation	8.9 (3.7)	n.n.	7 (1)	n.n.	0 (0)	0 (0)	0 (0)	54 (51)	53 (49)	0 (0)
6	Patient invited to MTC round after case discussion to explain recommendation	12.9 (5.2)	8.3 (5.5)	9 (2)	8 (2)	0 (0)	0 (0)	0 (0)	0 (0)	10 (23)	33 (77)
7	Patient invited to MTC round throughout their complete case discussion	7.1 (4.8)	4.7 (4.2)	12 (2)	13 (2)	7 (9.0)	70 (91)	0 (0)	0 (0)	0 (0)	0 (0)
Total		9.3 (4.5)	3.8 (3.5)	9 (4)	13 (3)	7 (2)	199 (48)	25 (6)	85 (21)	63 (15)	33 (8)

The observational data further reveal that case discussions in the patient's presence had a longer mean duration than those in the patient's absence (9 vs. 4 min) (see Table 3). MTCs with patient participation on average involved fewer persons (9 vs. 13 persons). Case discussions without patient participation predominantly took place in a theater‐style or U‐shape arrangement, and those with patients present, at round tables or in U‐shape arrangements. One hospital switches the seating arrangement to a round table when patients are invited.

### 3.3 What is the role of patients in the MTC?

Survey data at T1 from n = 82 patients who participated in the MTC show that most patients (62.2%) reported having been accompanied to the MTC meeting. Furthermore, 86.3% of patients reported that during the MTC, they had the opportunity to express their opinions regarding further treatment, and 61.0% reported having been involved in the treatment decision made in the MTC. All reports varied by hospital, as shown in Table 4.

**TABLE 4:** Descriptive results from survey items on patient reports of MTC participation (n = 82, T1) by hospital, n (%)

Item		Hospital				
		Total	4	5	6	7
Were you accompanied by someone (e.g., spouse, relative)?	Yes	51 (62.2)	14 (60.9)	28 (63.6)	7 (77.8)	2 (33.3)
	No	31 (37.8)	9 (39.1)	16 (36.4)	2 (22.2)	4 (66.7)
In the tumor conference, did you have the opportunity to express your opinion on further treatment?	Yes	69 (86.3)	17 (77.3)	38 (88.4)	9 (100.0)	5 (83.3)
	No	11 (13.8)	5 (22.7)	5 (11.6)	0 (0.0)	1 (16.7)
In the tumor conference, were you involved in the decision on your further treatment?	Yes	47 (61.0)	10 (45.5)	29 (69.0)	7 (87.5)	1 (20.0)
	No	30 (39.0)	12 (54.5)	13 (31.0)	1 (12.5)	4 (80.0)

**FIGURE 2:**
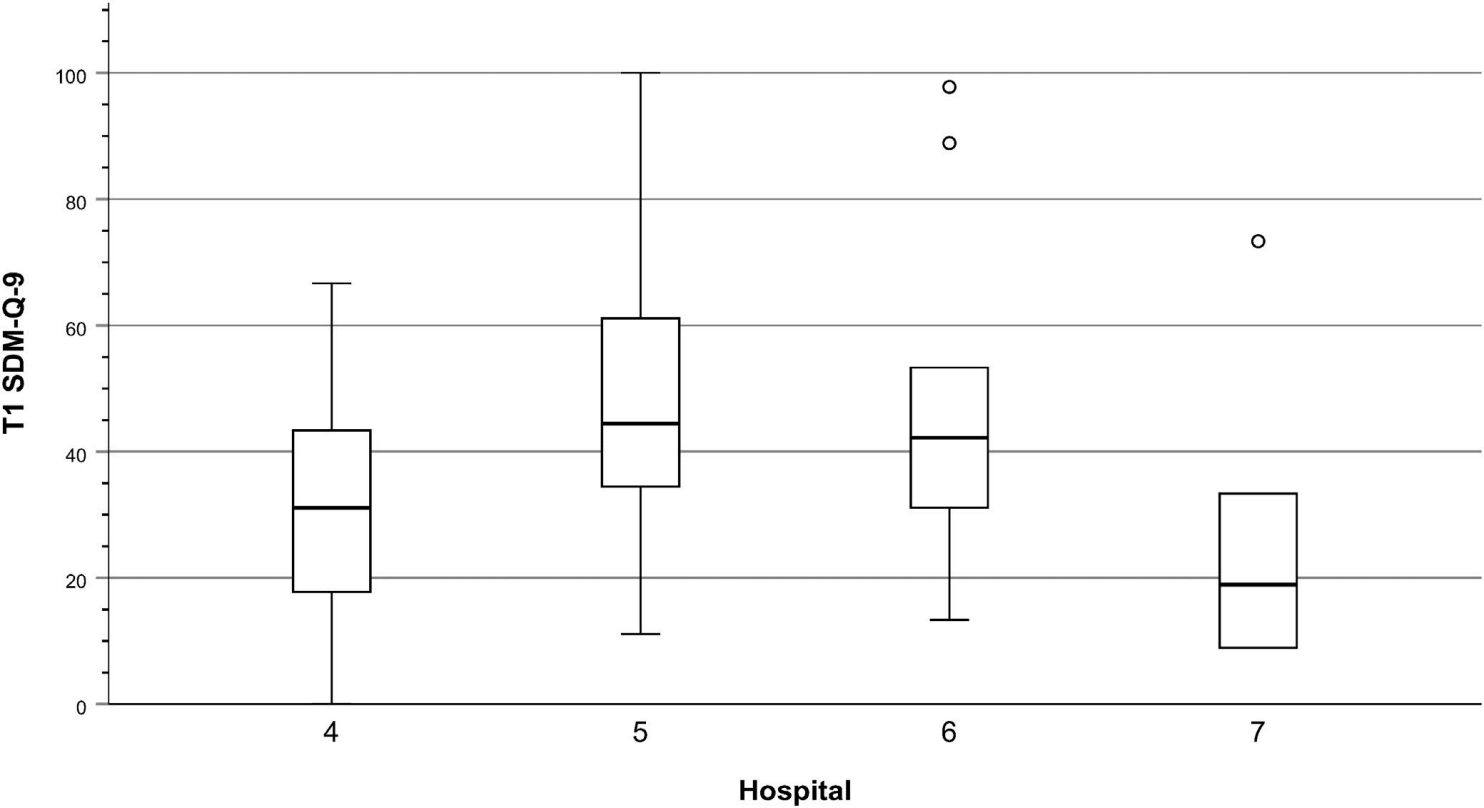
Box plot of the SDM‐Q‐9 by hospital with patient participation in MTC (n = 82, T1)

The median of the SDM‐Q‐9 was 37.8 but varied by hospital (see Figure 2). In hospital 7, where patients are present throughout the case discussion in a theater‐style hall, patients (n = 6) scored the lowest median of 18.9. In hospital 5, where a subgroup of MTC providers meets the patient in an extra room to summarize the case discussion and recommendation, patients (n = 43) scored the highest median of 44.4, with a large variance.

### 3.4 How do patients experience the MTC?

**FIGURE 3:**
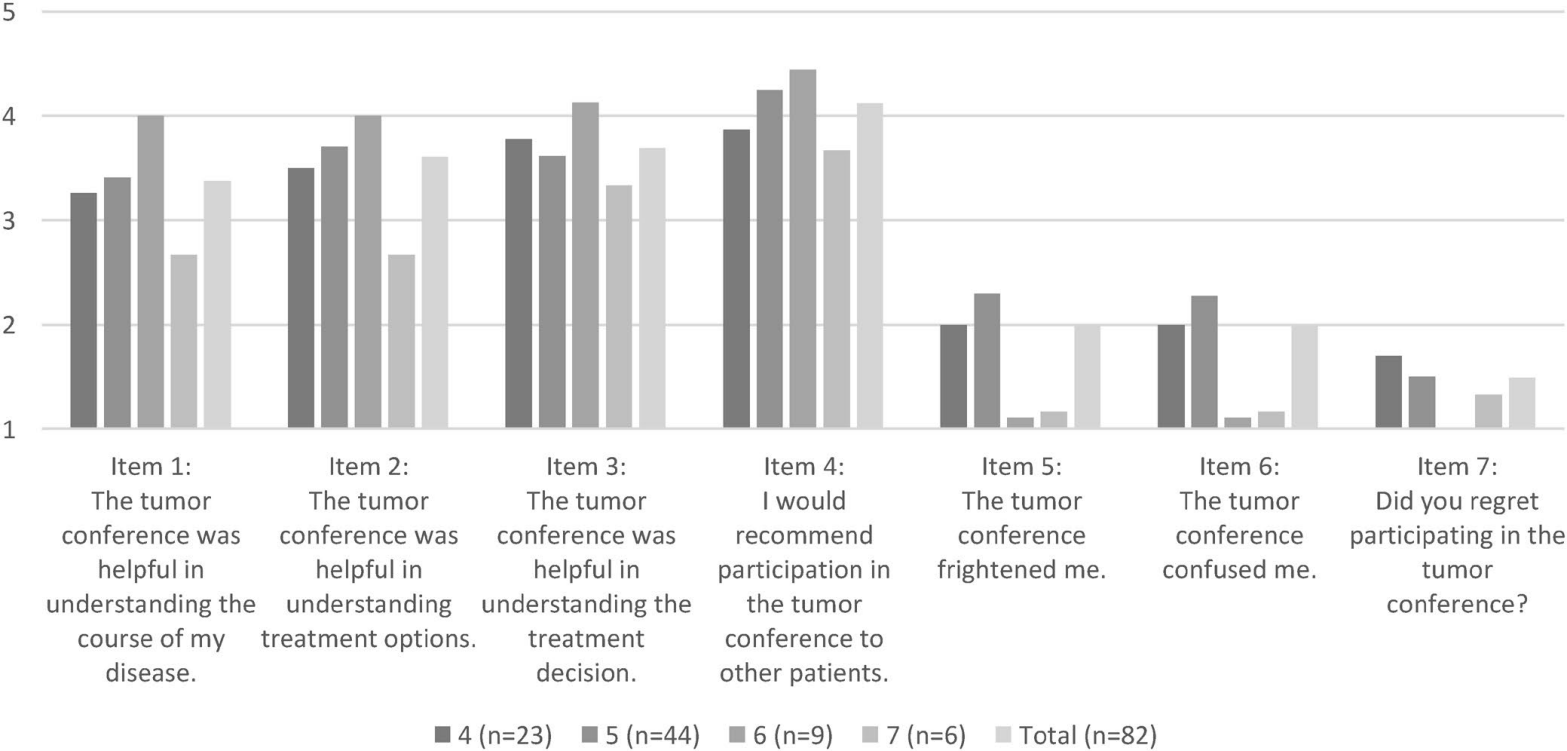
Bar chart of survey items on patients’ experiences with MTC participation (n = 82, T1); mean values by center (items 1–6: 1 = do not agree at all, 2 = do not agree, 3 = neutral, 4 = agree, 5 = completely agree; item 7: 1 = not at all, 2 = rather not, 3 = partly, 4 = rather yes, 5 = completely)

Survey data at T1 show that on average, patients reported their experience with the MTC as rather positive (see Figure 3). When asked how helpful the MTC was for understanding the course of the disease, the treatment options and the treatment decision, patients gave mean ratings of 3.4, 3.6, and 3.7, respectively, on a scale from 1 to 5. Willingness to recommend MTC participation to other patients had a mean score of 4.1. Fear and confusion resulting from participation in the MTC were both rated with a mean of 2.0. Regretting MTC participation scored a mean of 1.5. Figure 3 displays the variation by hospital, showing no consistent pattern of patients in specific hospitals having more positive or negative experiences.

## 4 DISCUSSION

As one of the first studies on patient participation in MTCs, we explored the implementation of patient participation as well as the patient's role and experience.

A main result of this explorative study is the high heterogeneity in implementation between the hospitals practicing patient participation. On the one end of the continuum, patients attend the complete case discussion for a mean of 7 min in a theater‐style hall with around 12 persons present. On the other end, patients attend a meeting for a mean of 9 min with around 7 MTC members in a smaller room at a round table after the larger MTC team has already discussed the case. Due to a lack of studies on MTC participation, our results can hardly be compared with the existing literature. However, a study on MTCs without patient participation in United Kingdom assumed that the seating arrangement determines the decision‐making quality within the team, although the intervention study did not confirm an effect.^23^ The rationale was that a U‐shape style will be more conducive to team interactions than a lecture hall style. Similarly, it can be assumed that the seating arrangement also determines interactions between the team and the patients participating in the MTC in our study. Our preliminary results suggest that a round table or a U‐shape style is the most frequently used seating arrangement when patients are present and one center even changes the seating arrangement from U‐shape to round table when patients are present. Thus, the effects of the seating arrangement on the interaction and on patient experiences with participation need to be further studied to be able to provide recommendations.

In the MTCs, patients seem to have an only partly active role. Whereas 9 out of 10 patients reported having had the opportunity to express their opinion on the treatment, only 6 out of 10 reported having been involved in the treatment decision; this confirms previous results from a larger survey.^9^ The SDM‐Q‐9 median of 37.8 on a scale of 0 to 100 demonstrates the patients’ rather passive role. This aligns well with previous qualitative findings^16^ that SDM implementation in MTCs with patient participation is limited by contextual constraints and healthcare providers’ attitudes.

The mainly positive but partly negative experiences with MTC participation described by patients confirm our previous finding that patients report a mixed picture of positive and negative cognitions and emotions.^9^ The vast majority of patients in our study would recommend patient participation in MTCs to other patients and did not regret participation, although a small number of patients would not recommend and did regret it. These results are in line with an Australian pilot study^14^ that found that the vast majority of patients found participation in MTCs helpful and recommendable. However, the proportion of patients with clearly negative experiences should not be neglected in research or in practice since these might be particularly vulnerable patients in need of more support. These patients may not benefit but may rather be harmed by attending MTCs due to the way it was implemented or due to them being emotionally too vulnerable for MTC participation. The identification of characteristics of patients who do not benefit from MTC participation is an important next step. Although the sample size in this study does not allow a detailed analysis of the subgroup of patients with negative experiences, supplementary bivariate correlation analyses of all patients with MTC participation (results not shown) indicate that patients with negative experiences have a significantly lower level of health literacy. The data did not show clear patterns of patients’ positive or negative experiences in relation to the ways the different hospitals implemented patient participation; this might be due to the low case numbers per hospital.

### 4.1 Strengths and limitations

This mixed methods study combined survey data with observational data, which is rarely done in health services research but offers many advantages, including better insight into how MTCs are implemented. The study itself provides even more data than can be presented here. Data from qualitative interviews, transcripts of MTCs, and patient‐reported outcomes have already been or will be additionally analyzed to gain comprehensive insight into the benefits and harms of patient participation in MTCs. Although this is the largest study on this topic so far, the numbers of hospitals and patients studied still limit its validity and do not allow stratification by subgroups of patients. However, it would be important to investigate which patients particularly benefit or are harmed by MTC participation. The high heterogeneity found between hospitals requires an even larger and possibly interventional study to fully determine the pros and cons of patient participation in MTCs. This explorative and unique observational study can build the basis for subsequent research. The sample characteristics of patients with and without patient participation did not substantially differ, except that all patients with participation were breast (and gynecological) cancer patients and none gynecological (and gastrointestinal) cancer patients. A slight tendency toward participating patients being younger, better educated, and more health literate can be seen. The findings are restricted to mainly breast cancer care, which means that it is currently unknown to what extent patient participation in MTCs is utilized in the care of other cancer entities in Germany and other countries. Moreover, the survey data analyzed here covered the patients’ experiences only directly after MTC participation, which can be subject to change over the course of disease and treatment. Also, audio and video data from this study on communication in MTCs are currently being analyzed and could provide additional valuable insights. Furthermore, qualitative interviews with participating patients are needed to complement the descriptions with patients’ interpretations.

## 5 CONCLUSIONS

Patient participation in MTCs seems to be a rare, but constant reality in breast cancer care in Germany. Most patients’ experiences with participating in the MTC were rather positive, but a small proportion of patients had negative experiences. Due to a lack of recommendations, hospitals implement patient participation in MTCs in various ways. So far, it is unknown which setting and procedures of MTC participation are beneficial for patients. However, existing evidence from research on communication in cancer care together with this exploratory study's findings can build the basis for developing recommendations for hospitals that invite their patients to MTCs. These recommendations would also be applicable to hospitals that do not regularly invite patients but may allow participation upon individual patient request. Particularly in these situations, providers without much experience may need support in implementing participation for the patient's benefit. Also, it should be mentioned that there might be alternative ways of ensuring patient‐centered MTCs, such as the participation of patient advocates (e.g., breast care nurses).^24^ The results of this explorative study do not yet warrant a conclusion on whether to recommend patient participation in MTCs, but they build the basis for subsequent comprehensive studies on this phenomenon. Regardless of its impact on patients, the feasibility of involving patients in MTCs is a limiting factor.

## 6 ETHICAL APPROVAL STATEMENT

The study received approval from the ethics committee of the Faculty of Medicine of the University of Cologne, Germany, and conforms with the Declaration of Helsinki.

## CONFLICT OF INTEREST

None of the authors has a conflict of interest.

## AUTHOR CONTRIBUTIONS

Lena Ansmann: Conceptualization, data curation, formal analysis, funding acquisition, investigation, methodology, project administration, resources, supervision, validation, visualization, writing––original draft, and writing––review and editing. Nicole Ernstmann: Conceptualization, funding acquisition, investigation, methodology, project administration, resources, supervision, and writing––review and editing. Christian Heuser, Annika Diekmann, and Barbara Schellenberger: Data curation, investigation, methodology, validation, and writing––review and editing. Claudia Biehl, Mahmoud Danaei, Christian Eichler, Dina Heinz, Andrea Hocke, Wolfram Malter, Badrig Melekian, Havva Metin, Alexander Mustea, Jenci Palatty, Uwe Peisker, and Ines Petschat: Investigation, and writing––review and editing.

## Data Availability

Data are available upon request.
